# The Glad Eye of Observation

**Published:** 1916-04-15

**Authors:** 


### THE MATRONS' AND SISTERS' DEPARTMENT.

### THE GLAD EYE OF OBSERVATION.

Some five years ago we started a series of articles, which have appeared from time to time since, on "Eminent Hospital Chairmen." In the course of the series we have had the privilege of bringing to the notice of the hospital world important service rendered for the sick and indirectly to the medical profession and to all ranks of people in these islands. It is not easy to find out when and where work of this invaluable character is being carried on under the voluntary system, and many a good man's and woman's labours have been performed for the public during a lifetime and passed out of recollection without adequate, if even the smallest, recognition. It is well periodically to take stock, as it were, of the whole hospital field, to note developments and changes, new movements, fresh departures, and the multitude of relatively small, but not unimportant, details which affect the happiness, the efficiency, and the work of all sections of the large army of workers who constitute what collectively may be defined as the staff of our hospitals.

To-day The Hospital has a wider and ever extending area of keen, knowledgeable, and interested readers, as our Letter-Box demonstrates. Not a few of them have most excellent critical qualities, and we wish that they would exercise them without stint, for every one of them who indulges this faculty helps to spread information and to promote the growth of efficiency. Others, again, take the keenest interest in their hospital, even when they are not directly connected with it, as they do indeed, and properly, in everything which is of value or interest to the community amongst whom they reside. Every man and woman who takes The Hospital has, therefore, an opportunity to become a person of the moment by taking a little trouble to secure the adequate recognition of individual work with which they may be familiar, and to invite the Editor's co-operation. There must be chairmen of many hospitals, several of the members of the staffs of hospitals, and devoted women who since the war have made a mark and won gratitude by their whole-hearted, unselfish, continuous labour in some section of the hospital field. We invite our readers to cultivate the glad eye of observation, to constitute themselves an illuminating centre of record in whatever branch of the hospital or nursing field they may occupy, by sending us such information as may make it possible to do justice to the eminent worker in their midst.

